# Mitochondrial disorders and ASD. Mechanisms of mitochondrial dysfunction in ASD

**DOI:** 10.1192/j.eurpsy.2023.285

**Published:** 2023-07-19

**Authors:** A. Sidenkova

**Affiliations:** Psychiatry, Ural State Medical University, Yekaterinburg, Russian Federation

## Abstract

**Introduction:**

ASD is a multifactorial disease. They arise from the interaction of various genetic and environmental factors. These factors affect specific neuronal circuits, oxidative stress, neuroinflammation, mitochondrial dysfunction. This disrupts the development of the nervous system, the formation of synapses, the connection between brain regions, and the size of the brain. Almost 80% of patients with ASD suffer from mitochondrial dysfunction. Therefore, mitochondrial dysfunction plays a crucial role in the pathogenesis of ASD.

**Objectives:**

Deficiency of adenosine-triphosphate (ATP) and abnormal levels of Reactive oxygen species (ROS) cause mitochondrial dysfunction in ASD. This leads to metabolic disorders, disorders of synaptic plasticity and disorders of the immune response

**Methods:**

The negative association between pathogenic mtDNA mutations and IQ is specific for children with ASD / MD. The overall prevalence of these abnormalities is 1.2 times higher in ASD / MD. ASD, researchers reaffirm that autistic probands carry the burden of mutations in mtDNA, especially mutations that are prone to deleterious effects on OXPHOS. According to a number of researchers, all children with ASD should be screened for MD, given: the high prevalence of abnormal markers of mitochondrial function in ASD compared with the control group; relatively high prevalence of MD in ASD; some children with ASD who have MD may be phenotypically indistinguishable from typical children with ASD; the potential clinical significance of MD in children with ASD.

**Results:**

According to a number of researchers, all children with ASD should be screened for MD, given: the high prevalence of abnormal markers of mitochondrial function in ASD compared with the control group; relatively high prevalence of MD in ASD; some children with ASD who have MD may be phenotypically indistinguishable from typical children with ASD; the potential clinical significance of MD in children with ASD.

**Conclusions:**

The pathophysiological mechanisms of ASD are multifactorial. They are largely unclear. But the mitochondrial hypothesis of the pathogenesis of ASD is being clarified. Mitochondrial dysfunction has been identified as a hallmark of diseased neurons in ASD patients, suggesting a critical role for mitochondrial dysfunction in the pathogenesis of ASD and allowing the development of ASD correction by normalizing mitochondrial functions.

**Disclosure of Interest:**

None Declared

